# Influence of cholestasis on portal vein embolization-induced hypertrophy of the future liver remnant

**DOI:** 10.1007/s00423-023-02784-w

**Published:** 2023-01-21

**Authors:** Xinwei Chang, Remon Korenblik, Bram Olij, Robrecht R. M. M. Knapen, Christiaan van der Leij, Daniel Heise, Marcel den Dulk, Ulf P. Neumann, Frank G. Schaap, Ronald M. van Dam, Steven W. M. Olde Damink

**Affiliations:** 1 Department of Surgery, Maastricht University Medical Center, Universiteitssingel 50, 6229 ER Maastricht, The Netherlands; 2 Department of Surgery, NUTRIM School of Nutrition and Translational Research in Metabolism, Maastricht University, Universiteitssingel 50, 6229 ER Maastricht, The Netherlands; 3 Breast Tumor Center, Sun Yat-Sen Memorial Hospital, Sun Yat-Sen University, Guangzhou, China; 4 GROW School for Oncology and Developmental Biology, Maastricht University, Maastricht, The Netherlands; 5 Department of Radiology and Nuclear Medicine, Maastricht University Medical Center, Maastricht, The Netherlands; 6 Department of General, Visceral and Transplant Surgery, University Hospital Aachen, Aachen, Germany

**Keywords:** Liver growth, Portal vein embolization, Cholestasis, Perihilar cholangiocarcinoma, Colorectal liver metastases

## Abstract

**Purpose:**

In the pre-clinical setting, hepatocellular bile salt accumulation impairs liver regeneration following partial hepatectomy. Here, we study the impact of cholestasis on portal vein embolization (PVE)-induced hypertrophy of the future liver remnant (FLR).

**Methods:**

Patients were enrolled with perihilar cholangiocarcinoma (pCCA) or colorectal liver metastases (CRLM) undergoing PVE before a (extended) right hemihepatectomy. Volume of segments II/III was considered FLR and assessed on pre-embolization and post-embolization CT scans. The degree of hypertrophy (DH, percentual increase) and kinetic growth rate (KGR, percentage/week) were used to assess PVE-induced hypertrophy.

**Results:**

A total of 50 patients (31 CRLM, 19 pCCA) were included. After PVE, the DH and KGR were similar in patients with CRLM and pCCA (5.2 [3.3–6.9] versus 5.7 [3.2–7.4] %, respectively, *p* = 0.960 for DH; 1.4 [0.9–2.5] versus 1.9 [1.0–2.4] %/week, respectively, *p* = 0.742 for KGR). Moreover, pCCA patients with or without hyperbilirubinemia had comparable DH (5.6 [3.0–7.5] versus 5.7 [2.4–7.0] %, respectively, *p* = 0.806) and KGR (1.7 [1.0–2.4] versus 1.9 [0.8–2.4] %/week, respectively, *p* = 1.000). For patients with pCCA, unilateral drainage in FLR induced a higher DH than bilateral drainage (6.7 [4.9–7.9] versus 2.7 [1.5–4.2] %, *p* = 0.012). C-reactive protein before PVE was negatively correlated with DH (*ρ* = − 0.539, *p* = 0.038) and KGR (*ρ* = − 0.532, *p* = 0.041) in patients with pCCA.

**Conclusions:**

There was no influence of cholestasis on FLR hypertrophy in patients undergoing PVE. Bilateral drainage and inflammation appeared to be negatively associated with FLR hypertrophy. Further prospective studies with larger and more homogenous patient cohorts are desirable.

**Supplementary Information:**

The online version contains supplementary material available at 10.1007/s00423-023-02784-w.

## Introduction

Most major hepatectomies are performed to achieve a complete margin-free resection with a curative intention, in patients with hepatobiliary tumors. However, in patients undergoing resection, a small future liver remnant (FLR) volume is associated with a higher incidence of post-hepatectomy liver failure and death [1, 2]. In order to achieve a safe liver resection, the FLR volume must be > 30% in uncompromised livers, > 40% in chemotherapy-damaged or steatotic livers, and > 50% in cirrhotic livers [3, 4]. In practice, liver resections are commonly performed in patients with underlying liver disease, and an insufficient FLR volume may preclude surgery [5].

The current standard procedure to induce hypertrophy of the non-embolized liver segments is portal vein embolization (PVE), where the aim is to increase FLR volume prior to resection [6]. PVE has been associated with an increase in FLR volume of 20–50% within 3–7 weeks [7, 8]. Studies have shown that PVE-induced hypertrophy of the FLR has a beneficial effect on reducing the rates of liver failure and death after major liver resection [2, 9]. PVE induces insufficient hypertrophy of the FLR (i.e., failure to proceed to hepatectomy) in approximately 30% patients with colorectal liver metastasis (CRLM) [10], which might be linked to the formation of collateral blood vessels in embolized segments. For this reason, new techniques are being investigated to induce hypertrophy of the FLR. A novel procedure, i.e., simultaneously performed portal and hepatic vein embolization (PVE/HVE), which aims at preventing formation of collaterals in embolized segments, showed better FLR hypertrophy than PVE alone and had comparable postoperative survival [11–15]. PVE/HVE is currently being further evaluated by the international DRAGON trials collaborative [16, 17].

Besides more effective hypertrophy-inducing techniques, investigations are also being conducted to identifying those factors that negatively or positively affect cell growth in the non-embolized liver lobe [18, 19]. In that respect, cholestasis is frequently present in patients with hepatobiliary tumors, especially in patients with perihilar cholangiocarcinoma (pCCA). Previous studies have demonstrated that during cholestasis excessive hepatic bile salt levels occur, which impair the proliferative capacity of hepatocytes [20, 21]. By contrast, stimulation of bile salt signaling by agonistic activation of the bile salt receptor farnesoid X receptor (FXR) was found to promote liver growth in a rabbit PVE model [19]. A wealth of animal studies have stressed the importance of maintained bile salt homeostasis in the proper progression of liver growth following partial hepatectomy [22–24].

A few studies have examined the impact of cholestasis on FLR hypertrophy in patients undergoing PVE, but their findings have been inconclusive [8, 25]. One recent study, for example, reported similar hypertrophy of the FLR in patients with a primary biliary malignancy and normal or elevated (i.e., ≥ 50 µmol/L) serum bilirubin [25]. The aim of the present study was to test the hypothesis that cholestasis impairs FLR hypertrophy in patients undergoing PVE. To this end, PVE-induced FLR hypertrophy was analyzed based on serum bilirubin levels. In addition, we investigated the associations between routine serum biochemical tests and/or biliary drainage, and FLR hypertrophy.

## Materials and methods

### Study design

This study is a bi-center retrospective cohort study. Adult patients (≥ 18 years) with pCCA or CRLM were considered who had undergone PVE prior to (extended) right hepatectomy between January 01, 2016 and December 31, 2019 in the Maastricht University Medical Center (the Netherlands) or in the Uniklinik Aachen (Germany). Patients who had missing liver computed tomography (CT) scans, partial resection of the FLR between PVE and liver volume assessment, or only inadvertent drainage of the tumor-bearing segments were excluded. This study was approved by the institutional review boards of both centers (METC2019-1375 and EK 434/19).

### Assessment of liver volume

Since all included patients were planned for (extended) right hepatectomy, liver segments II and III were consistently part of the FLR. The volume of these segments, which can be measured accurately and with consistency due to the *ligamentum teres hepatis* landmark for the virtual cut, was taken as the measure of FLR. Measured total liver volumes (mTLV), tumor volumes (TV), and FLR volumes were determined from routine CT scans both prior to and after PVE. In addition, standardized total liver volume (sTLV) taking into account body surface area was calculated according to Vauthey et al. [26]. If multiple PVE procedures had been attempted to induce sufficient FLR volume gain, the liver volumetry after the last PVE was used. The diameters of the left and right hepatic bile duct were measured on pre-embolization scans, with bile duct dilation indicative for obstructive cholestasis. Additionally, the degree of bile duct dilation was assessed visually as no, mild, moderate, or severe. CT scans were locally de-identified and volumetry was performed using Syngo.via Liver Analysis, Siemens Healthineers. All volumetries were performed by an investigator (XC), checked by another investigator (RK), and both were proctored by a local interventional radiologist (CL).

### Definitions and data collection

FLR increase was defined as the difference in FLR volume (FLRV) before (FLRV1) and after PVE (FLRV2). Measured FLR share (mFLR) was calculated as: ${\mathrm{mFLR}}\mathrm{=}\frac{\mathrm{FLRV}}{\mathrm{mTLV-TV}}$ [5, 27]. In addition, standardized FLR (sFLR) share was calculated as $\mathrm{sFLR}=\frac{\mathrm{FLRV}}{\mathrm{sTLV}}$ [1]. %Hypertrophy was defined as $\frac{\mathrm{FLRV}2-\mathrm{FLRV}1}{\mathrm{FLRV}1}$. The degree of hypertrophy (DH) was defined as the difference in FLR share: $\mathrm{DH }=\mathrm{ mFLR}2-\mathrm{mFLR}1$ or $\mathrm{DH}=\mathrm{sFLR}2-\mathrm{sFLR}1$. Kinetic growth rate (KGR) was calculated as $\mathrm{KGR}=\frac{\mathrm{DH}}{t}$, where *t* is the time (in weeks) elapsed between PVE and the CT volumetry after PVE. Tumor volume was subtracted from the FLRV in cases of presence of tumor in the FLR.

The following main clinical and laboratory variables were retrieved from the patient files: tumor type, histological status of liver parenchyma, occurrence of drainage prior to PVE, and presence of cholangitis prior to PVE. Routine serum biochemistry parameters (bilirubin, albumin, international normalized ratio [INR], gamma-glutamyl transferase [GGT], alkaline phosphatase [ALP], alanine aminotransferase [ALT], aspartate aminotransferase [AST], C-reactive protein [CRP], and white blood cell count) were gathered prior to PVE and—if applicable—prior to biliary drainage. Bilirubin level ≥ 50.0 µmol/L was used as a clinical marker of cholestasis. Confirmation bias was prevented by having a separate researcher (BO) collect the data from the Electronic Health Records. All data were gathered in CASTOR EDC, Amsterdam, a Good Clinical Practice-accepted online data capturing and monitoring system.

### Statistical analyses

All statistical analyses were performed using SPSS 24.0 (IBM, Armonk, New York, USA). Data are presented as median [interquartile range] or frequency (percentage) when appropriate. For continuous data, differences between two groups were compared using the Mann–Whitney *U* test. Serum biochemistry parameters, assessed sequentially in patients with pCCA before drainage and before PVE, were compared using a Wilcoxon signed rank test. Categorical data was compared using chi-square test or Fisher’s exact test as appropriate. Correlations were evaluated by Spearman rank correlation coefficients (*ρ*). Statistical significance was considered at *p* < 0.05.

## Results

### Patient characteristics

A total of 50 patients (CRLM, *n* = 31; pCCA, *n* = 19) were enrolled and their characteristics are shown in Table 1. Patients diagnosed with CRLM had a higher median Charlson comorbidity index than those with pCCA and had larger tumors, but were otherwise similar. All patients underwent embolization of the right portal branch, with the intention to undergo (extended) right hemihepatectomy. Four patients received an extended segment IV embolization (Table 1). The technical success of the PVE procedure (successful obstruction of the right portal branch) was achieved in all patients. Two patients with CRLM had more than one PVE procedure. Tumors were present in liver segments II/III in eight patients with CRLM. Twenty-nine out of thirty-one patients with CRLM received chemotherapy before PVE, whereas none of the patients with pCCA received chemotherapy.

**Table 1:** Baseline characteristics of participants

Characteristics	CRLM (*n* = 31)	pCCA (*n* = 19)	*p* value
Age (years)	65 [56–73]	70 [67–76]	0.070
Gender			0.276
Female	7 (23%)	7 (37%)	
Male	24 (77%)	12 (63%)	
BMI (kg/m^2^)	25.2 [22.8–28.4]	23.4 [22.3–26.3]	0.448
Diabetes	6 (19%)	4 (21%)	1.000
Charlson comorbidity index	8 [7–9]	6 [5–8]	< *0.001*
Tumor volume (mL)	37.0 [9.8–165.3]	2.0 [0.3–3.5]	< *0.001*
Nontumoral liver parenchyma^†^			0.946
Normal	18 (62%)	11 (65%)	
Fibrosis	5 (17%)	2 (12%)	
Cirrhosis	1 (4%)	1 (6%)	
Steatosis	5 (17%)	3 (17%)	
Cholangitis	0 (0%)	3 (16%)	
Bismuth-Corlette classification			
I	-	1 (5%)	
II	-	2 (11%)	
IIIA	-	5 (26%)	
IIIB	-	1 (5%)	
IV	-	10 (53%)	
Chemotherapy			< *0.001*
No	2 (6%)	19 (100%)	
FOLFOX/XELOX	23 (74%)		
FOLFIRI/FOLFOXIRI	5 (16%)		
Other	1 (4%)		
Type of biliary drainage			< *0.001*
No	31 (100%)	2 (11%)	
PTBD	-	2 (11%)	
EBD	-	9 (47%)	
Both	-	6 (31%)	
Side of biliary drainage			
Unilateral	-	11 (65%)	
Bilateral	-	6 (35%)	
Hepatic bile duct diameter (mm)			
Left	< 3.0	6.3 [5.3–10.0]	< *0.001*
Right	< 3.0	7.3 [5.4–10.7]	< *0.001*
Hepatic bile duct dilation (left or right)			< *0.001*
No	31 (100%)	0 (0%)	
Mild + moderate	-	8 (42%)	
Severe	-	11 (58%)	
Type of PVE			0.629
Right PVE	29 (94%)	17 (90%)	
Right PVE extended to segment IV	2 (6%)	2 (10%)	

Data are presented as median [interquartile range] or frequency (percentage). Significant *p*-values are presented in italic

*CRLM* colorectal liver metastasis; *pCCA* perihilar cholangiocarcinoma; *BMI* body mass index; *PTBD* percutaneous transhepatic biliary drainage; *EBD* endoscopic biliary drainage; *FLR* future liver remnant; *PVE* portal vein embolization

^†^Histological information is not available for two patients in each group

None of the patients with CRLM was drained, whereas seventeen out of nineteen patients with pCCA underwent biliary drainage prior to PVE. Of those seventeen patients, eleven received unilateral drainage of the FLR, with the remaining six receiving simultaneous drainage of the right hepatic lobe.

### pCCA patients remain cholestatic at the time of PVE

To assess the efficacy of biliary drainage in patients with pCCA, serum biochemistry parameters prior to and after biliary drainage were compared (Supplementary Table S1). Thirteen out of seventeen patients had hyperbilirubinemia before drainage, with bilirubin data missing for one patient. Serum bilirubin levels were reduced 2.6-fold after drainage, but remained above the normal range in 10 patients. After drainage, ALT levels were decreased, and there was a trend towards a reduction of AST levels (*p* = 0.026 and *p* = 0.087, respectively). Prior to the PVE procedure, patients with pCCA had higher serum levels of bilirubin, GGT, and ALP than patients with CRLM (Supplementary Table S1), reflecting cholestatic liver injury and its incomplete resolution by drainage.

### Liver growth after PVE

The median time from the initial PVE procedure to volumetric assessment post-PVE was 22 [19–29] days and 20 [16–22] days in patients with CRLM and pCCA, respectively (*p* = 0.141, Table 2). The absolute FLR volume increased from 263 [208–327] to 363 [322–439] mL after PVE in patients with CRLM, and from 326 [220–437] to 394 [363–542] mL in patients with pCCA (Supplementary Fig. S1A). Accordingly, the FLR% increased significantly after PVE in both groups (Supplementary Fig. S1B).

**Table 2:** Assessment of FLR hypertrophy after PVE between patients with CRLM and pCCA

Variable	CRLM (*n* = 31)	pCCA (*n* = 19)	*p* value
Number of days after PVE	22 [19–29]	20 [16–22]	0.141
FLR increase (mL)	88 [53–139]	120 [49–165]	0.522
FLR increase per day (mL/day)	3.5 [1.9–6.9]	4.5 [2.4–8.8]	0.299
%Hypertrophy	30 [19–59]	38 [14–65]	0.897
%Hypertrophy per week	9.2 [6.0–18.3]	10.6 [5.2–19.8]	0.667
DH (%) using mFLR	5.2 [3.3–6.9]	5.7 [3.2–7.4]	0.960
KGR (% per week) using mFLR	1.4 [0.9–2.5]	1.9 [1.0–2.4]	0.742
DH (%) using sFLR	5.2 [3.5–7.7]	8.4 [3.4–11.4]	0.212
KGR (% per week) using sFLR	1.4 [1.0–2.5]	2.3 [1.2–4.5]	0.117
TV after PVE (mL)	85.0 [20.5–203.0]	2.0 [1.0–3.0]	< *0.001*

Data are presented as median [interquartile range]. Significant *p*-values are presented in italic

*CRLM* colorectal liver metastasis; *pCCA* perihilar cholangiocarcinoma; *PVE* portal vein embolization; *FLR* future liver remnant; *DH* degree of hypertrophy; *KGR* kinetic growth rate; *TV* tumor volume

The absolute FLR volume increase and %hypertrophy were not different in CRLM and pCCA groups (*p* = 0.522 and *p* = 0.897, respectively) (Table 2). In addition, patients with CRLM and pCCA had similar DH using mFLR (5.2 [3.3–6.9] versus 5.7 [3.2–7.4] %, respectively, *p* = 0.960) or sFLR (5.2 [3.5–7.7] versus 8.4 [3.4–11.4] %, respectively, *p* = 0.212). Likewise, KGR after PVE was similar in these groups, employing either mFLR (1.4 [0.9–2.5] versus 1.9 [1.0–2.4] %/week, respectively, *p* = 0.742) or sFLR (1.4 [1.0–2.5] versus 2.3 [1.2–4.5] %/week, respectively, *p* = 0.117) as basis for calculations (Table 2).

As patients with CRLM had a higher Charlson comorbidity index, a sensitivity analysis was performed, which was done by 1:1 matching based on age, gender, BMI, Charlson comorbidity index, cirrhosis, and presence of diabetes. The characteristics of the matched patients (*n* = 10 in each group) are presented in Supplementary Table S2. Both DH and KGR were still comparable between patients with CRLM and pCCA, and not dependent on use of measured or standardized volumes (DH-mFLR: 5.9 [3.8–7.8] versus 5.8 [1.8–7.1] %, respectively, *p* = 0.597; DH-sFLR: 6.4 [3.9–8.4] versus 7.8 [1.8–10.8] %, respectively, *p* = 0.880; KGR-mFLR: 1.6 [0.9–2.8] versus 1.6 [0.5–2.4] %/week, respectively, *p* = 0.597; KGR-sFLR: 2.1 [0.8–3.4] versus 1.7 [0.7–3.8] %/week, respectively, *p* = 1.000).

### Cholestasis does not affect PVE-induced liver growth in pCCA

**Fig. 1:**
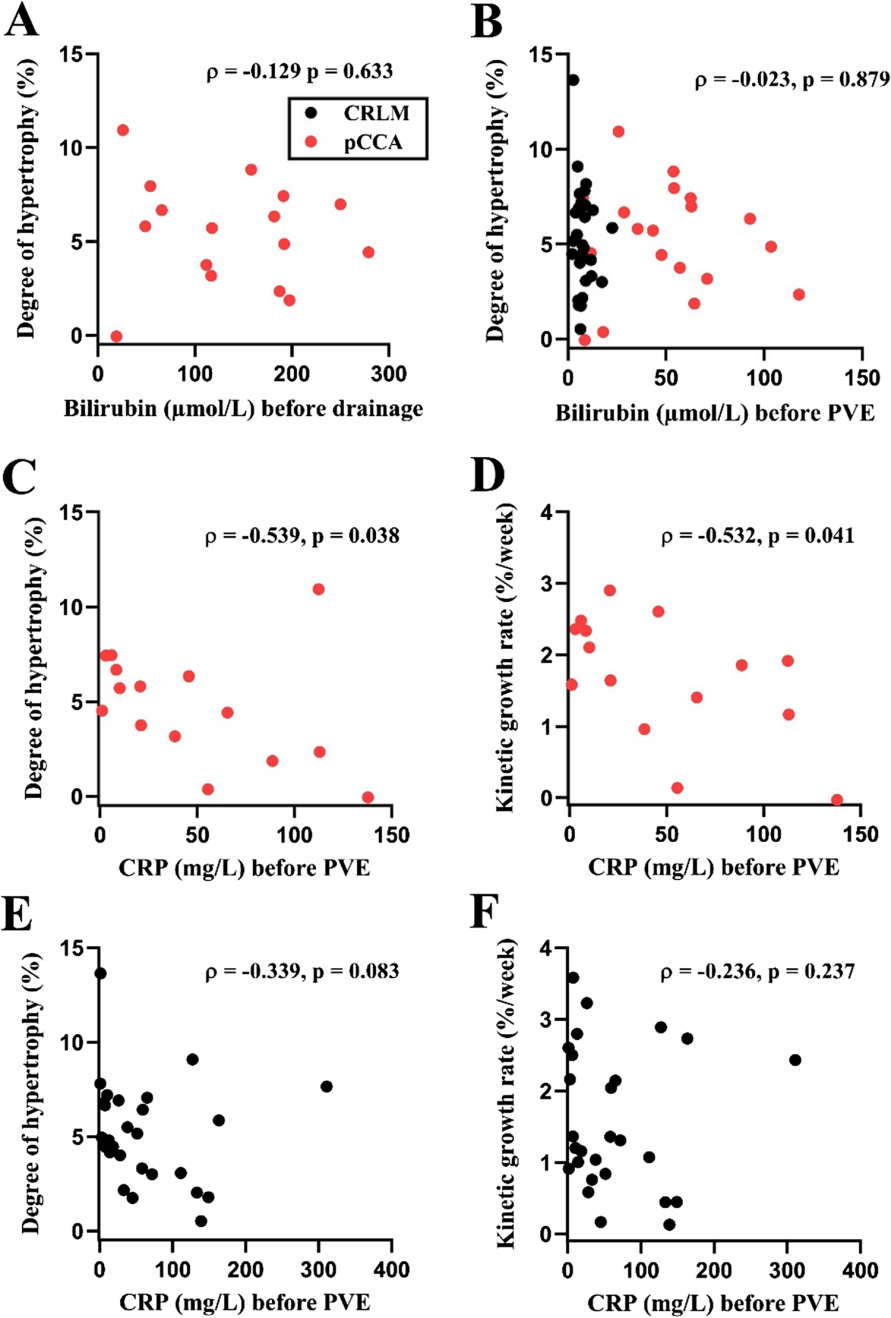
Serum CRP negatively correlates with hypertrophy of FLR in patients with pCCA. Correlations between degree of hypertrophy of FLR and serum bilirubin levels before drainage (**A**) and before PVE (**B**). Correlations between serum CRP levels and degree of hypertrophy and kinetic growth rate of FLR in patients with pCCA (**C** and **D**, respectively) and CRLM (**E** and **F**, respectively). mFLR was used for assessing hypertrophy of FLR. Correlations were assessed using Spearman’s rank test. CRLM, colorectal liver metastasis; pCCA, perihilar cholangiocarcinoma; CRP, C-reactive protein

After biliary drainage, ten patients with pCCA still had hyperbilirubinemia. These patients had comparable DH and KGR to patients with bilirubin levels lower than 50 µmol/L (DH-mFLR: 5.6 [3.0–7.5] versus 5.7 [2.4–7.0] %, respectively, *p* = 0.806; DH-sFLR: 9.9 [3.9–12.8] versus 5.5 [1.9–11.3] %, respectively, *p* = 0.288; KGR-mFLR: 1.7 [1.0–2.4] versus 1.9 [0.8–2.4] %/week, respectively, *p* = 1.000; KGR-sFLR: 3.1 [1.1–4.7] versus 1.7 [0.7–3.5] %/week, respectively, *p* = 0.288) (Table 3). Spearman correlation analyses also showed that serum bilirubin levels before drainage (pCCA group: *ρ* = − 0.129, *p* = 0.633) or before PVE (entire cohort: *ρ* = − 0.023, *p* = 0.879) were not correlated with the DH by using mFLR, with similar results employing sFLR (Fig. 1A-B, Supplementary Table S4). Likewise, serum bilirubin levels before drainage (pCCA group: *ρ* = + 0.097, *p* = 0.72 using mFLR) or before PVE (entire cohort: *ρ* = + 0.148, *p* = 0.321 using mFLR) were unrelated to KGR.

**Table 3:** Assessment of FLR hypertrophy after PVE in pCCA patients with and without hyperbilirubinemia

Variable	Hyperbilirubinemia group (*n* = 10)	Without hyperbilirubinemia group (*n* = 9)	*p* value
Age (years)	70 [67–72]	70 [64–76]	0.563
Gender			0.650
Female	3 (30%)	4 (44%)	
Male	7 (70%)	5 (56%)	
BMI (kg/m^2^)	22.8 [22.0–26.1]	25.6 [23.2–27.0]	0.102
Charlson comorbidity index	6 [5–8]	7 [4–8]	0.934
Cholangitis	2 (20%)	1 (11%)	1.000
Right PVE extended to segment IV	1 (10%)	1 (11%)	1.000
Serum biochemistry parameters before PVE			
Bilirubin (µmol/L)	63.9 [56.4–95.5]	25.8 [10.0–39.4]	< *0.001*
GGT (U/L)	535 [333–1029]	339 [106–753]	0.191
ALP (U/L)	369 [271–725]	255 [128–420]	0.083
CRP (mg/L)	42.1 [16.4–94.7]	20.6 [7.0–89.0]	0.637
Number of days after PVE	22 [16–26]	20 [17–22]	0.652
FLR increase (mL)	131 [68–189]	91 [28–144]	0.253
FLR increase per day (mL/day)	6.2 [2.2–11.0]	4.1 [1.5–7.2]	0.288
%Hypertrophy	56 [15–67]	30 [8–51]	0.191
%Hypertrophy per week	16.4 [6.0–27.0]	10.4 [2.8–16.1]	0.221
DH (%) using mFLR	5.6 [3.0–7.5]	5.7 [2.4–7.0]	0.806
KGR (% per week) using mFLR	1.7 [1.0–2.4]	1.9 [0.8–2.4]	1.000
DH (%) using sFLR	9.9 [3.9–12.8]	5.5 [1.9–11.3]	0.288
KGR (% per week) using sFLR	3.1 [1.1–4.7]	1.7 [0.7–3.5]	0.288

Data are presented as median [interquartile range]. Significant *p*-values are presented in italic

*pCCA* perihilar cholangiocarcinoma; *PVE* portal vein embolization; *FLR* future liver remnant; *GGT* gamma-glutamyl transferase; *ALP* alkaline phosphatase; *CRP* C-reactive protein; *DH* degree of hypertrophy; *KGR* kinetic growth rate

In the pCCA group, there were no differences in DH and KGR between patients with mild-moderate bile duct dilation (*n* = 8) and those with severe bile duct dilation (*n* = 11) (DH-mFLR: 6.0 [4.5–8.5] versus 4.5 [2.4–7.0] %, respectively, *p* = 0.283; DH-sFLR: 8.9 [3.9–16.2] versus 8.4 [2.3–10.5] %, respectively, *p* = 0.322; KGR-mFLR: 2.0 [1.1–2.6] versus 1.6 [1.0–2.3] %/week, respectively, *p* = 0.509; KGR-sFLR: 2.2 [1.4–4.7] versus 2.7 [0.9–4.5] %/week, respectively, *p* = 0.804). Additionally, the diameter of bile ducts was not correlated with the DH and KGR (*ρ* = − 0.396, *p* = 0.084 using mFLR; *ρ* = − 0.269, *p* = 0.252 using mFLR, respectively). In the CRLM group, the DH and KGR were similar in patients with cirrhosis or liver fibrosis (*n* = 6) and those with normal background liver (*n* = 18) (DH-mFLR: 6.4 [3.0–9.3] versus 4.6 [3.0–6.4] %, respectively, *p* = 0.205; DH-sFLR: 6.6 [3.1–9.9] versus 5.0 [3.4–7.5] %, respectively, *p* = 0.549; KGR-mFLR: 1.8 [0.9–2.6] versus 1.2 [0.8–2.1] %/week, respectively, *p* = 0.351; KGR-sFLR: 1.4 [0.8–3.1] versus 1.3 [0.9–2.2] %/week, respectively, *p* = 0.739).

**Fig. 2:**
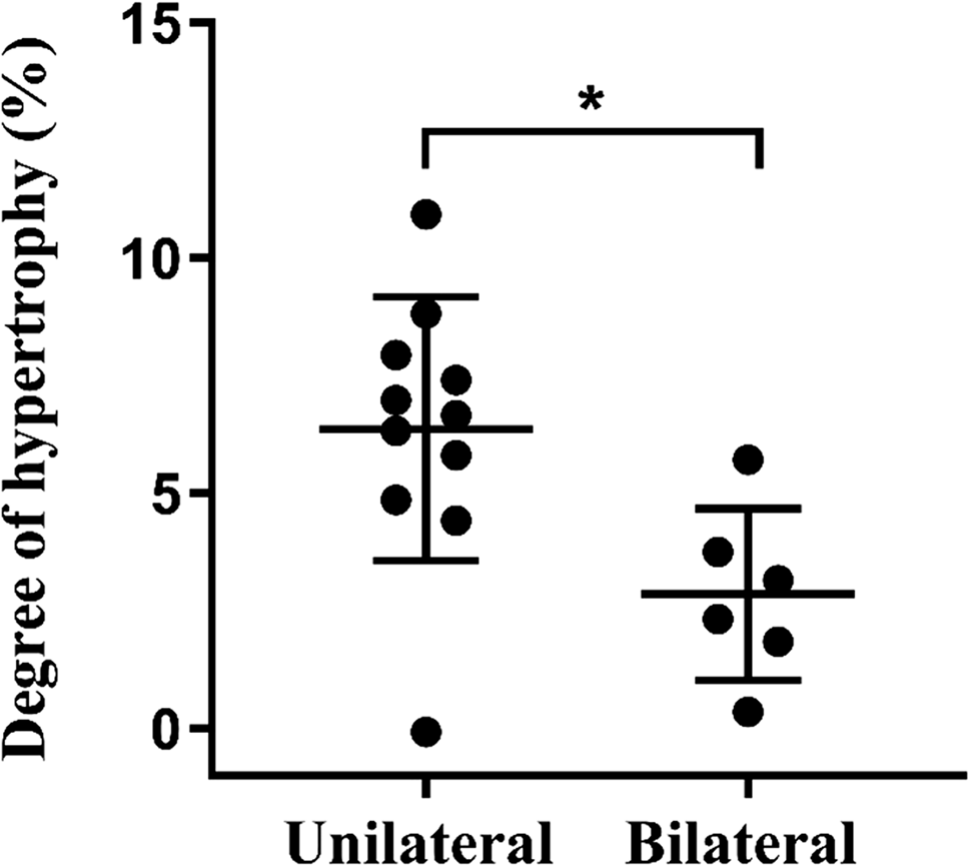
Degree of hypertrophy of the future liver remnant is higher in patients with unilateral biliary drainage. Seventeen out of nineteen patients with perihilar cholangiocarcinoma received biliary drainage for relief of cholestasis prior to portal vein embolization. The future liver remnant (FLR) was drained in patients receiving unilateral drainage (*n* = 11). mFLR was used to assess the degree of hypertrophy. Mann–Whitney *U* test was used to compare degree of hypertrophy of FLR between two groups. Asterisks indicate significance level: *p < 0.05

The FLR hypertrophy response was analyzed according to the side (unilateral, bilateral) of biliary drainage prior to PVE in seventeen patients with pCCA. Patients with unilateral drainage (*n* = 11) had a higher DH than those with bilateral drainage (*n* = 6) (6.7 [4.9–7.9] versus 2.7 [1.5–4.2] %, *p* = 0.012 using mFLR; 10.7 [8.4–14.2] versus 2.8 [1.6–5.7] %, *p* = 0.012 using sFLR) (Fig. 2, Supplementary Fig. S2). The characteristics of pCCA patients with unilateral and bilateral biliary drainage were similar (Supplementary Table S3**)**.

### Correlations between routine blood tests and FLR hypertrophy

A Spearman correlation analysis was performed to evaluate associations between serum biochemistry and FLR hypertrophy parameters. Serum CRP levels before PVE were negatively correlated with the DH (*ρ* = − 0.401, *p* = 0.009 using mFLR), and showed a strong tendency to be negatively correlated with KGR (*ρ* = − 0.300, *p* = 0.054 using mFLR). Subgroup analysis revealed that the inverse relationships between CRP and FLR hypertrophy were apparent in patients with pCCA (DH: *ρ* = − 0.539, *p* = 0.038; KGR: *ρ* = − 0.532, *p* = 0.041, respectively both using mFLR) (Fig. 1C-D), but not in patients with CRLM (DH: *ρ* = − 0.339, *p* = 0.083; KGR: *ρ* = − 0.236, *p* = 0.237, respectively both using mFLR) (Fig. 1E-F). Additionally, serum albumin levels before PVE were positively correlated with the DH (*ρ* = + 0.301, *p* = 0.045 using mFLR). All correlations between serum biochemistry parameters and FLR hypertrophy are summarized in Supplementary Table S4.

## Discussion

This study has evaluated whether cholestasis affects PVE-induced hypertrophy of the future liver remnant. The main findings are that the degree of hypertrophy and kinetic growth rate of the FLR are similar in patients with CRLM and pCCA, and in pCCA patients with or without hyperbilirubinemia. Neither serum bilirubin levels before drainage, nor before PVE, were correlated with indices of FLR hypertrophy. Patients with pCCA receiving unilateral biliary drainage of the FLR had a higher DH than those with bilateral drainage. Elevated inflammatory response was associated with impaired liver growth in patients with pCCA.

In previous studies, cholestasis has been associated with poor regenerative capacity of the liver after partial hepatectomy, with toxicity due to bile salt overload in hepatocytes serving as an underlying mechanism [28]. Patients with pCCA, characterized by cholestasis in one or both liver halves due to biliary obstruction, have been shown to have a high 90-day mortality rate of up to 14% after partial liver resection, despite routine biliary decompression prior to surgery [29]. In the present study, we observed similar FLR hypertrophy after PVE, in patients with CRLM and pCCA, which is in line with recent findings elsewhere [30]. The majority (89%) of pCCA patients here received drainage prior to PVE. Nine out of nineteen patients with pCCA had serum bilirubin levels lower than 50 µmol/L at the time of PVE. These nine patients had comparable DH and KGR with pCCA patients who had bilirubin levels higher than 50 µmol/L. Moreover, serum bilirubin either before drainage or before PVE were not correlated with the DH and KGR. The similar FLR hypertrophy in cholestatic and non-cholestatic patients suggests that the growth-stimulating effect of PVE outweighs any potential negative effects of bile salt accumulation.

Yim et al. [25] reported that hyperbilirubinemia (used cut-off: ≥ 51 µmol/L) at the time of PVE had no effect on FLR hypertrophy, which is coincident with our findings. In this study, only patients with primary biliary malignancy were included [25]. In our study, we investigated the role of cholestasis on PVE-induced liver hypertrophy not only by comparing hyperbilirubinemia with non-hyperbilirubinemia in patients with pCCA, but also by comparing patients with CRLM to pCCA. Moreover, we analyzed the associations between biliary drainage, serum biochemistry markers and FLR hypertrophy, which could stimulate further research in the interaction between inflammation (CRP) and FLR hypertrophy.

A previous study conducted by Kasai et al. [8] reported that maximum bilirubin level at the time of PVE was a negative predictive factor of FLR hypertrophy in a cohort of 59 patients. However, our study showed that bilirubin levels before PVE were not correlated with FLR hypertrophy. The reason of the incongruent results may be the different extent of hyperbilirubinemia between two studies. The mean maximum bilirubin level before PVE was 126 µmol/L in the cohort studied by Kasai et al. [8]. In our study, the median bilirubin level before PVE was 9 µmol/L for the entire cohort, and 53.8 µmol/L for patients with pCCA. In addition, the liver parenchyma histological background and use of chemotherapy were different between these two studies. Therefore, all these factors could lead to inconsistent findings regarding FLR volume augmentation after PVE.

Biliary drainage is widely used to decompress the biliary tree and improve cholestatic status before liver resection [31, 32]. In our study, seventeen of nineteen patients with pCCA underwent biliary drainage before PVE. Remarkably, the DH was higher in those patients with unilateral drainage of the FLR than in those with bilateral drainage. Our findings are thus consistent with the study of Ishizawa et al., who observed a higher hypertrophy ratio and bilirubin clearance rate of the FLR after unilateral drainage of the FLR group than the bilateral drainage group [33]. In our cohort, patients with bilateral drainage had insufficient improvement of cholestasis upon initial biliary drainage. Of note, the occurrence of cholangitis appeared to be higher in the bilateral drainage group (2 out 6 = 33% versus 1 out of 11 = 9%), although low event rates do not allow statistical back-up. It is nonetheless tempting to speculate that cholangitis and associated inflammation contributed to impaired liver hypertrophy in bilaterally drained patients. It should be noted that serum CRP, which was found to negatively correlate with DH, was not affected by the side of drainage.

Our study has indicated a negative relation between serum CRP levels before PVE and the DH and KGR, especially in patients with pCCA. To the best of our knowledge, this is the first study to reveal the association of an inflammation marker and FLR growth in clinical patients. Notably, serum CRP levels were 1.7-fold higher after biliary drainage in patients with pCCA, albeit this did not reach statistical significance (*p* = 0.167). The tentative CRP elevation might have been due to the retrograde route through the proximal small intestine in the case of endoscopic biliary drainage (EBD). In support of this notion, 15 out of 17 (88%) drained patients received an EBD or EBD + PTBD (percutaneous transhepatic biliary drainage) procedure. Our study was in line with findings of Yokoyama et al. [34], who showed that the daily non-embolized liver lobe increase rate was significantly decreased in patients with cholangitis, indicating the negative influence of inflammation on FLR growth after PVE.

The question of which molecular pathway could underlie the observed relationship between CRP and FRL hypertrophy remains unclear. However, it is known that cytokines (e.g., interleukin-8) induced by inflammation can stimulate hepatic infiltration of neutrophils [35]. As part of innate immune defenses, neutrophils produce reactive oxygen species and hypochlorous acid that cause cytotoxicity [36]. Experimental and human studies have shown that periportal neutrophil infiltration is positively correlated with the degree of liver damage [36, 37], which is recognized as having a negative impact on liver hypertrophy. Serum CRP alterations may have promise as a surrogate marker of PVE-induced FLR hypertrophy in patients with pCCA.

Certain limitations have to be acknowledged. For example, the study’s retrospective design makes it impossible to avoid selection bias when including patients. In addition, the sample size of the present cohort is rather small, which means some events appear rare, such as cholangitis, PTBD procedure, and non-drainage of the liver in pCCA. As such, it is impossible to conduct a multivariate logistic regression to assess the predictive value of these factors on FLR hypertrophy. Furthermore, nine patients had small metastases in segment II or III, which might have impacted PVE-induced hypertrophy. Nevertheless, the volume of the small metastases was excluded when analyzing FLR volumes. Besides Charlson comorbidity index, the use of chemotherapy prior to PVE was different between CRLM and pCCA groups, and these factors could influence liver hypertrophy. A meta-analysis conducted by Soykan et al. showed that chemotherapy had no effects on the hypertrophy of FLR [38]. Several retrospective studies reported contradictory associations of chemotherapy drugs with PVE-induced liver hypertrophy [1, 8, 39, 40], but well-designed prospective studies are warranted to confirm if chemotherapy has an influence on FLR hypertrophy.

Besides bilirubin, bile salts are another indicator reflecting cholestasis, although not one routinely assayed in clinical chemistry. Due to the unavailability of serum samples, bile salt levels could not be assessed. Bile salts are involved in liver regeneration after partial hepatectomy via activation of the nuclear bile salt receptor FXR [22]. FXR plays a central role in maintaining bile salt homeostasis, and regulating hepatocyte cell cycle progression [23]. Moreover, we have previously demonstrated that FXR agonist obeticholic acid accelerates liver growth following PVE in rabbits [19]. Bile salt levels at 5 h after PVE predict FLR volume after 3 weeks in patients scheduled for resection [18]. It would therefore be worthwhile including serum bile salt measurements in larger prospective studies to address the influence of cholestasis on PVE-induced hypertrophy in pCCA patients.

## Conclusion

Cholestasis was found to have no influence on hypertrophy of the FLR in patients undergoing PVE. Bilateral drainage and inflammation might negatively affect the FLR hypertrophy. Further prospective studies with larger and more homogenous patient cohorts are needed.

### Supplementary Information

Below is the link to the electronic supplementary material.

Supplementary file1 (DOCX 219 KB)

## Data Availability

Data available on request from the authors.

## Abbreviations

FLR: Future liver remnant
PVE: Portal vein embolization
CRLM: Colorectal liver metastases
PVE/HVE: Combined portal and hepatic vein embolization
pCCA: Perihilar cholangiocarcinoma
FXR: Farnesoid X receptor
CT: Computed tomography
DH: Degree of hypertrophy
KGR: Kinetic growth rate
ALP: Alkaline phosphatase
GGT: Gamma-glutamyl transferase
ALT: Alanine aminotransferase
AST: Aspartate aminotransferase
INR: International normalized ratio
CRP: C-reactive protein
TLV: Total liver volume
BMI: Body mass index
EBD: Endoscopic biliary drainage
PTBD: Percutaneous transhepatic biliary drainage
