# Supplementary material for: Influence of cholestasis on portal vein embolization-induced hypertrophy of the future liver remnant
Source: Langenbecks Arch Surg. 2023 Jan 21;408(1):54. doi: 10.1007/s00423-023-02784-w (PMC9867667; doi:10.1007/s00423-023-02784-w)
Supplement: Supplementary file 1 — Supplementary file1 (DOCX 219 KB) [file 423_2023_2784_MOESM1_ESM.docx]

**Influence of cholestasis on portal vein embolization-induced hypertrophy of the future liver remnant**

Langenbeck's Archives of Surgery

Xinwei Chang^*^, Remon Korenblik^*^, Bram Olij, Robrecht R.M.M. Knapen, Christiaan van der Leij, Daniel Heise, Marcel den Dulk, Ulf Neumann, Frank G. Schaap, Ronald van Dam, Steven W.M. Olde Damink

**Correspondence:** Dr. Xinwei Chang, MD, PhD, Department of Surgery, Maastricht University Medical Center, NUTRIM School of Nutrition and Translational Research in Metabolism, Maastricht University, Universiteitssingel 50, 6229 ER Maastricht, The Netherlands. E-mail: xinweichang@hotmail.com

| \| **Supplementary Table S1** Serum biochemistry tests in patients with pCCA and CRLM \| \| \| \| \| \| \| --- \| --- \| --- \| --- \| --- \| --- \| \| Parameters \| pCCA (n=19) \| \| p value **^†^** \| CRLM (n=31) \| p value **^‡^** \| \| Before drainage \| Before PVE \| Before PVE \| \| Bilirubin (µmol/L) \| 137.8 [57.0-191.9] \| 53.8 [25.8-64.5] \| **0.001** \| 6.9 [5.2-8.9] \| **<0.001** \| \| Albumin (g/L) \| 36.3 [33.3-40.8] \| 35.0 [27.7-37.3] \| 0.188 \| 36.0 [30.3-42.0] \| 0.246 \| \| INR \| 1.02 [0.98-1.12] \| 1.02 [1.00-1.08] \| 0.364 \| 1.01 [0.97-1.10] \| 0.732 \| \| GGT (U/L) \| 735 [297-1048] \| 434 [225-878] \| 0.300 \| 77 [36-185] \| **<0.001** \| \| ALP (U/L) \| 438 [249-571] \| 318 [208-463] \| 0.173 \| 121 [86-155] \| **<0.001** \| \| ALT (U/L) \| 133 [76-310] \| 103 [58-127] \| **0.026** \| 50 [27-150] \| 0.235 \| \| AST (U/L) \| 124 [78-169] \| 74 [42-103] \| 0.087 \| 39 [26-94] \| 0.053 \| \| CRP (mg/L) \| 22.7 [11.2-73.8] \| 38.5 [8.2-88.6] \| 0.131 \| 38.0 [10.1-111.0] \| 0.684 \| \| WBC count (10^9^/L) \| 8.7 [5.3-11.1] \| 8.2 [6.0-10.0] \| 0.155 \| 6.5 [6.2-8.9] \| 0.271 \|   Data are presented as median [interquartile range].  **^†^** p values were calculated for comparison between “before drainage” and “before PVE” values, within patients with pCCA.  **^‡^** p values were calculated for comparison of “before PVE” values between pCCA and CRLM groups.  pCCA, perihilar cholangiocarcinoma; CRLM, colorectal liver metastasis; INR, international normalized ratio; GGT, gamma-glutamyl transferase; ALP, alkaline phosphatase; ALT, alanine aminotransferase; AST, aspartate aminotransferase; CRP, C-reactive protein; WBC, white blood cell.  **Supplementary Table S2** Characteristics of 10 matched patients with CRLM and pCCA | | | | |
| --- | --- | --- | --- | --- | --- | --- | --- | --- | --- | --- | --- | --- | --- | --- | --- | --- | --- | --- | --- | --- | --- | --- | --- | --- | --- | --- | --- | --- | --- | --- | --- | --- | --- | --- | --- | --- | --- | --- | --- | --- | --- | --- | --- | --- | --- | --- | --- | --- | --- | --- | --- | --- | --- | --- | --- | --- | --- | --- | --- | --- | --- | --- | --- | --- | --- | --- | --- | --- | --- | --- | --- | --- | --- |
| Characteristics | CRLM (n=10) | pCCA (n=10) | p value |  |
| Age (years) | 61 [47-71] | 70 [69-73] | 0.079 |  |
| Gender |  |  | 1.000 |  |
| Female | 4 (40%) | 5 (50%) |  |  |
| Male | 6 (60%) | 5 (50%) |  |  |
| BMI (kg/m^2^) | 24.6 [23.1-29.0] | 25.7 [23.1-26.5] | 0.705 |  |
| Diabetes | 2 (20%) | 2 (20%) | 1.000 |  |
| Charlson comorbidity index | 8 [6-9] | 8 [7-9] | 0.969 |  |
| Cirrhosis | 0 (0%) | 0 (0%) | 1.000 |  |

Data are presented as median [interquartile range] or frequency (percentage). CRLM, colorectal liver metastasis; pCCA, perihilar cholangiocarcinoma; BMI, body mass index.

| **Supplementary Table S3** Characteristics of 17 pCCA patients with biliary drainage | | | |
| --- | --- | --- | --- |
| Variable | Unilateral drainage (n=11) | Bilateral drainage (n=6) | p value |
| Age (years) | 70 [67-72] | 73 [69-77] | 0.359 |
| Gender |  |  | 1.000 |
| Female | 5 (46%) | 2 (33%) |  |
| Male | 6 (54%) | 4 (67%) |  |
| BMI (kg/m^2^) | 24.6 [22.6-27.0] | 22.8 [22.1-27.8] | 0.615 |
| Diabetes | 4 (36%) | 0 (0%) | 0.237 |
| Charlson comorbidity index | 6 [5-8] | 7 [5-9] | 0.721 |
| Cholangitis | 1 (9%) | 2 (33%) | 0.515 |
| Bismuth-Corlette classification |  |  | 0.266 |
| Ⅰ | 1 (9%) | 0 (0%) |  |
| Ⅱ | 2 (18%) | 0 (0%) |  |
| ⅢA | 3 (27%) | 1 (17%) |  |
| ⅢB | 1 (9%) | 0 (0%) |  |
| Ⅳ | 4 (37%) | 5 (83%) |  |
| Serum biochemistry tests before PVE |  |  |  |
| Bilirubin (µmol/L) | 53.8 [28.6-62.9] | 60.8 [36.9-82.7] | 0.421 |
| Albumin (g/L) | 35.5 [30.0-38.5] | 30.5 [22.4-35.3] | 0.158 |
| INR | 1.01 [0.95-1.12] | 1.02 [1.01-1.09] | 0.359 |
| GGT (U/L) | 719 [414-1083] | 342 [281-612] | 0.108 |
| ALP (U/L) | 379 [220-662] | 291 [242-376] | 0.396 |
| ALT (U/L) | 115 [60-152] | 66 [46-98] | 0.090 |
| AST (U/L) | 82 [42-205] | 84 [46-103] | 0.914 |
| CRP (mg/L) | 45.6 [8.2-112.5] | 47.0 [18.2-94.7] | 0.775 |
| WBC count (10^9^/L) | 8.8 [6.2-12.5] | 7.1 [4.9-9.3] | 0.289 |
| Number of days after PVE | 22 [17-35] | 17 [12-20] | 0.086 |

Data are presented as median [interquartile range] or frequency (percentage). pCCA, perihilar cholangiocarcinoma; BMI, body mass index; PVE, portal vein embolization; INR, international normalized ratio; GGT, gamma-glutamyl transferase; ALP, alkaline phosphatase; ALT, alanine aminotransferase; AST, aspartate aminotransferase; CRP, C-reactive protein; WBC, white blood cell.

| **Supplementary Table S4** Correlations between serum biochemistry parameters and FLR hypertrophy | | | | | | | | | |
| --- | --- | --- | --- | --- | --- | --- | --- | --- | --- |
| Parameters | DH | | | |  | KGR | | | |
|  | Using mFLR | | Using sFLR | |  | Using mFLR | | Using sFLR | |
|  | ρ | p value | ρ | p value |  | ρ | p value | ρ | p value |
| ***Entire cohort (n=50)*** |  |  |  |  |  |  |  |  |  |
| Parameters before PVE |  |  |  |  |  |  |  |  |  |
| Bilirubin (µmol/L) | -0.023 | 0.879 | 0.116 | 0.437 |  | 0.148 | 0.321 | 0.280 | 0.056 |
| Albumin (g/L) | 0.301 | **0.045** | 0.134 | 0.381 |  | 0.110 | 0.471 | -0.011 | 0.941 |
| INR | -0.189 | 0.193 | -0.193 | 0.184 |  | -0.120 | 0.412 | -0.103 | 0.482 |
| GGT (U/L) | 0.139 | 0.345 | 0.283 | 0.051 |  | 0.074 | 0.618 | 0.236 | 0.106 |
| ALP (U/L) | -0.021 | 0.892 | 0.330 | **0.029** |  | -0.007 | 0.963 | 0.289 | 0.057 |
| ALT (U/L) | -0.018 | 0.911 | 0.104 | 0.521 |  | -0.154 | 0.342 | -0.041 | 0.801 |
| AST (U/L) | -0.120 | 0.442 | 0.053 | 0.737 |  | -0.039 | 0.803 | 0.077 | 0.621 |
| CRP (mg/L) | -0.401 | **0.009** | -0.157 | 0.322 |  | -0.300 | 0.054 | -0.101 | 0.524 |
| WBC count (10^9^/L) | -0.072 | 0.640 | 0.026 | 0.867 |  | -0.083 | 0.589 | 0.024 | 0.874 |
| ***CRLM patients*** ***(n=31)*** |  |  |  |  |  |  |  |  |  |
| Parameters before PVE |  |  |  |  |  |  |  |  |  |
| Bilirubin (µmol/L) | -0.082 | 0.678 | -0.195 | 0.320 |  | 0.215 | 0.273 | 0.113 | 0.567 |
| Albumin (g/L) | 0.176 | 0.370 | -0.022 | 0.912 |  | 0.066 | 0.738 | -0.136 | 0.490 |
| INR | 0.013 | 0.945 | 0.079 | 0.674 |  | 0.034 | 0.855 | 0.129 | 0.488 |
| GGT (U/L) | 0.113 | 0.560 | 0.143 | 0.458 |  | 0.116 | 0.548 | 0.137 | 0.478 |
| ALP (U/L) | -0.275 | 0.164 | 0.005 | 0.978 |  | -0.130 | 0.519 | 0.010 | 0.960 |
| ALT (U/L) | -0.312 | 0.129 | -0.284 | 0.170 |  | -0.329 | 0.108 | -0.366 | 0.072 |
| AST (U/L) | -0.304 | 0.140 | -0.189 | 0.367 |  | -0.077 | 0.713 | -0.052 | 0.805 |
| CRP (mg/L) | -0.339 | 0.083 | -0.030 | 0.882 |  | -0.236 | 0.237 | 0.022 | 0.913 |
| WBC count (10^9^/L) | -0.151 | 0.443 | -0.045 | 0.820 |  | -0.108 | 0.584 | -0.042 | 0.830 |
| ***pCCA patients (n=19)*** |  |  |  |  |  |  |  |  |  |
| Parameters before drainage |  |  |  |  |  |  |  |  |  |
| Bilirubin (µmol/L) | -0.129 | 0.633 | 0.003 | 0.991 |  | 0.097 | 0.721 | 0.159 | 0.557 |
| Albumin (g/L) | 0.302 | 0.340 | -0.060 | 0.854 |  | 0.256 | 0.422 | 0.046 | 0.888 |
| INR | -0.502 | 0.057 | -0.516 | **0.049** |  | -0.203 | 0.469 | -0.283 | 0.306 |
| GGT (U/L) | 0.565 | **0.023** | 0.341 | 0.196 |  | -0.032 | 0.905 | -0.047 | 0.863 |
| ALP (U/L) | 0.391 | 0.134 | 0.624 | **0.010** |  | 0.256 | 0.339 | 0.350 | 0.184 |
| ALT (U/L) | 0.289 | 0.296 | 0.064 | 0.820 |  | -0.021 | 0.940 | -0.029 | 0.919 |
| AST (U/L) | 0.407 | 0.149 | 0.310 | 0.281 |  | -0.086 | 0.771 | -0.068 | 0.817 |
| CRP (mg/L) | -0.218 | 0.455 | -0.222 | 0.446 |  | -0.345 | 0.227 | -0.402 | 0.154 |
| WBC count (10^9^/L) | -0.135 | 0.661 | -0.275 | 0.363 |  | -0.030 | 0.922 | -0.256 | 0.399 |
| Parameters before PVE |  |  |  |  |  |  |  |  |  |
| Bilirubin (µmol/L) | -0.105 | 0.668 | 0.186 | 0.446 |  | -0.025 | 0.920 | 0.228 | 0.348 |
| Albumin (g/L) | 0.503 | **0.039** | 0.347 | 0.172 |  | 0.415 | 0.098 | 0.211 | 0.416 |
| INR | -0.513 | **0.030** | -0.600 | **0.008** |  | -0.469 | **0.050** | -0.522 | **0.026** |
| GGT (U/L) | 0.481 | **0.037** | 0.600 | **0.007** |  | -0.016 | 0.949 | 0.242 | 0.318 |
| ALP (U/L) | 0.353 | 0.165 | 0.694 | **0.002** |  | 0.172 | 0.510 | 0.471 | 0.057 |
| ALT (U/L) | 0.579 | **0.024** | 0.589 | **0.021** |  | 0.271 | 0.328 | 0.421 | 0.118 |
| AST (U/L) | 0.298 | 0.229 | 0.328 | 0.183 |  | 0.037 | 0.884 | 0.103 | 0.683 |
| CRP (mg/L) | -0.539 | **0.038** | -0.346 | 0.206 |  | -0.532 | **0.041** | -0.307 | 0.265 |
| WBC count (10^9^/L) | 0.032 | 0.903 | 0.034 | 0.896 |  | -0.225 | 0.384 | -0.186 | 0.474 |

Spearman correlation analyses were performed to evaluate associations between serum biochemistry parameters and FLR hypertrophy. DH, Degree of hypertrophy; KGR, kinetic growth rate; INR, international normalized ratio; GGT, gamma-glutamyl transferase; ALP, alkaline phosphatase; ALT, alanine aminotransferase; AST, aspartate aminotransferase; CRP, C-reactive protein; WBC, white blood cell; CRLM, colorectal liver metastasis; pCCA, perihilar cholangiocarcinoma.


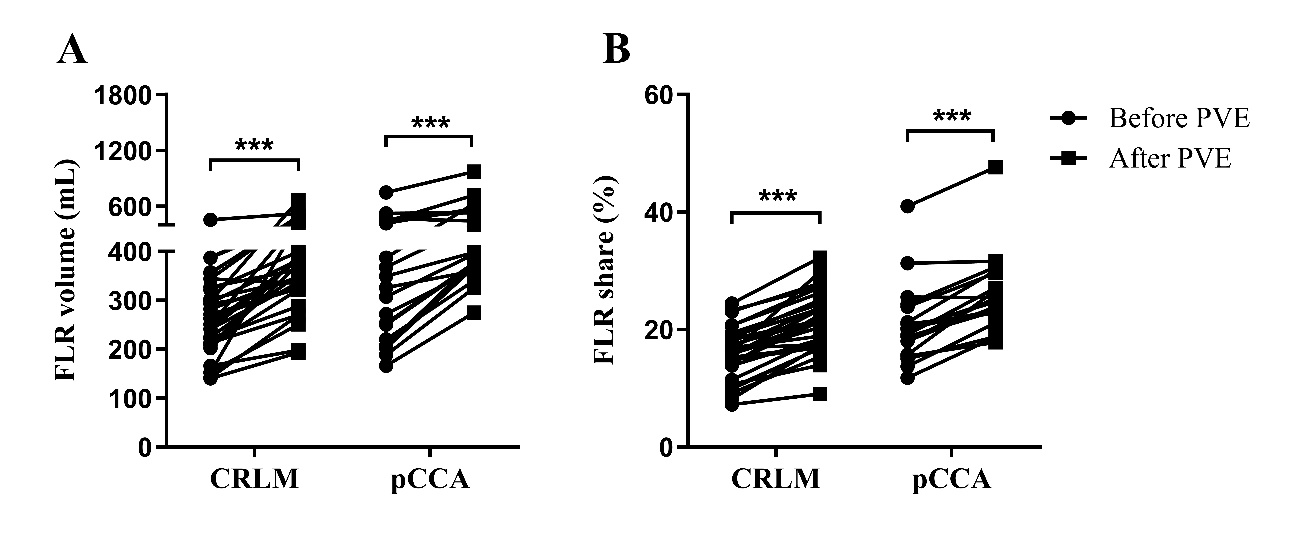


**Supplementary Fig. S1** Volumetric expansion of the future liver remnant by PVE in individual patients. The increase of absolute future liver remnant (FLR) volume (A) and measured FLR share (B) after portal vein embolization (PVE) in patients with colorectal liver metastasis (CRLM) and perihilar cholangiocarcinoma (pCCA). Asterisks indicate significance level: ^***^p<0.001.


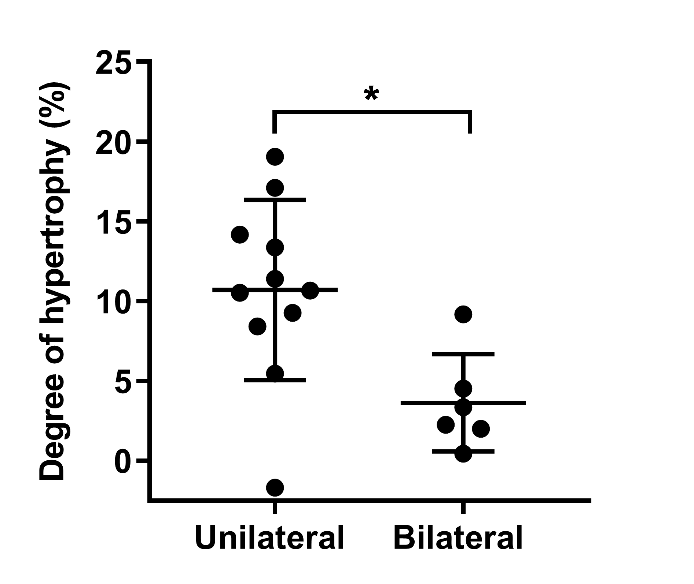


**Supplementary Fig. S2** Degree of hypertrophy of the future liver remnant is higher in patients with unilateral biliary drainage. sFLR was used to assess the degree of hypertrophy. Mann-Whitney U test was used to compared degree of hypertrophy of FLR between two groups. Asterisks indicate significance level: ^*^p<0.05.
